# ‘Indirect’ challenges from science to clinical practice

**DOI:** 10.3402/ecrj.v3.31096

**Published:** 2016-02-22

**Authors:** Sandra D. Anderson

**Affiliations:** Sydney Medical School, Central Clinical School, University of Sydney, Sydney, NSW, Australia

**Keywords:** exercise, eucapnic hyperpnoea, hypertonic saline, dry powder mannitol, disodium cromoglycate, indirect challenges, airway hyperresponsiveness

## Abstract

Indirect challenges act to provoke bronchoconstriction by causing the release of endogenous mediators and are used to identify airway hyper-responsiveness. This paper reviews the historical development of challenges, with exercise, eucapnic voluntary hyperpnoea (EVH) of dry air, wet hypertonic saline, and with dry powder mannitol, that preceded their use in clinical practice. The first challenge developed for clinical use was exercise. Physicians were keen for a standardized test to identify exercise-induced asthma (EIA) and to assess the effect of drugs such as disodium cromoglycate. EVH with dry air became a surrogate for exercise to increase ventilation to very high levels. A simple test was developed with EVH and used to identify EIA in defence force recruits and later in elite athletes. The research findings with different conditions of inspired air led to the conclusion that loss of water by evaporation from the airway surface was the stimulus to EIA. The proposal that water loss caused a transient increase in osmolarity led to the development of the hypertonic saline challenge. The wet aerosol challenge with 4.5% saline, provided a known osmotic stimulus, to which most asthmatics were sensitive. To simplify the osmotic challenge, a dry powder of mannitol was specially prepared and encapsulated. The test pack with different doses and an inhaler provided a common operating procedure that could be used at the point of care. All these challenge tests have a high specificity to identify currently active asthma. All have been used to assess the benefit of treatment with inhaled corticosteroids. Over the 50 years, the methods for testing became safer, less complex, and less expensive and all used forced expiratory volume in 1 sec to measure the response. Thus, they became practical to use routinely and were recommended in guidelines for use in clinical practice.

‘Indirect challenges act by causing the release of endogenous mediators that cause airway smooth muscle to contract, with or without effect in inducing microvascular leakage. Because the responses to these challenges are modified or even completely inhibited by inhaled steroids, the airway response to these challenges may be a close reflection of active airway inflammation’ (1). This review covers some of the scientific history of the development of the ‘indirect’ challenges that have become established in clinical practice for bronchial provocation testing. These are exercise, eucapnic voluntary hyperpnoea of dry air, a wet aerosol of hypertonic saline, and a dry powder aerosol of mannitol (2).

## Introduction

In the 1960s, there were several events that ultimately led to the development of ‘indirect’ challenge tests to identify airway hyperresponsiveness (AHR). The first was the recognition that children with asthma could have an ‘attack’ provoked by exercise, a non-immunological stimulus (3, 4). This ‘attack’ was identified by a transient increase in airways resistance after exercise. The terms first used to describe it were exercise-induced asthma (EIA) (5), exercise-induced bronchospasm (6), and exercise-induced bronchoconstriction (7).

Another event was the commercial release in 1968 of the drug disodium cromoglycate (DSCG) given by inhalation as a dry powder (8). The mode of action of DSCG, from *in vitro* studies, suggested that it stabilised the mast cell membrane, inhibiting the immunological (via IgE) release of histamine in response to inhaled allergen. DSCG had no direct action on airway smooth muscle (8), yet it was very effective in preventing both the early- and late-phase airway response to inhaled allergen (9, 10). At the time, allergen inhalation challenge, also an indirect challenge was used on a case-by-case basis particularly to identify sensitivity to an occupational exposure. These allergen challenges were difficult to standardize and unsuitable for use to evaluate DSCG, either in children or in large population studies. DSCG, however, had also been reported to inhibit EIA in adults (11, 12). The potential for children with EIA to benefit from DSCG was quickly recognised and investigators focused on developing a ‘standardized’ exercise test and on identifying an easy to measure index for change in lung function (13–16).

## Exercise

### In quest of a suitable protocol

An early protocol to identify EIA in children, in the USA, was 5 min of cycle ergometer exercise of sufficient intensity to reach a heart rate of 180 bpm (13, 17). The maximum decrease or fall in forced expiratory volume in 1 sec (FEV_1_) recorded in the 20 min after exercise, expressed as a percentage of the pre-exercise value, was used to measure the response. Eighteen (72%) asthmatics had a % fall ≥10% in FEV_1_. The response was characterised as mild <10% fall, moderate 10–25%, and severe >25%. Only four normal non-asthmatic subjects were tested and none had a sustained fall in FEV_1_.

As running was identified as more potent for provoking EIA than cycling, protocols were also developed for exercising on a motorised treadmill (18, 19). Peak expiratory flow (PEF) was often used, rather than FEV_1_, because it could be measured quickly during exercise to document a rise as well as a fall after exercise (20). The coefficient of variation for repeated measures of PEF was 4.1%. For running exercise, the upper limit for the % fall (mean+2SD) in PEF in normal non-asthmatic children was found to be 10% (21).

**Fig. 1:**
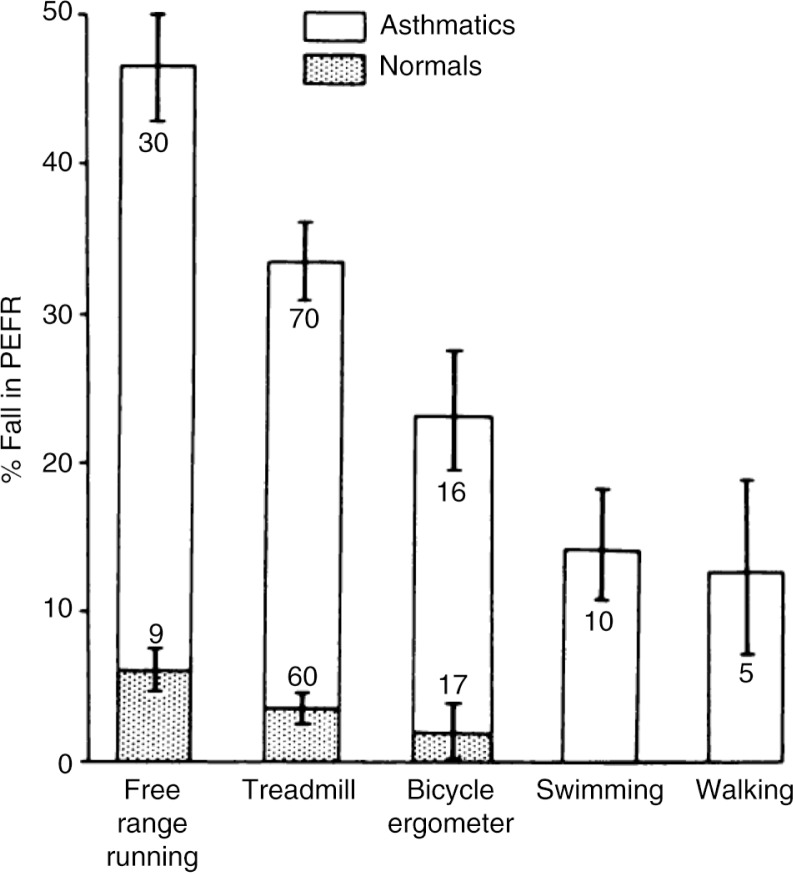
Exercise-induced asthma expressed as a % fall from baseline PEFR in groups of asthmatic and normal subjects. Although the individual subjects were not all the same in each group they were all working at the same relative work load. The numbers indicate the number of subjects. The bars indicate±SEM. Reproduced with permission from (14).

**Fig. 2:**
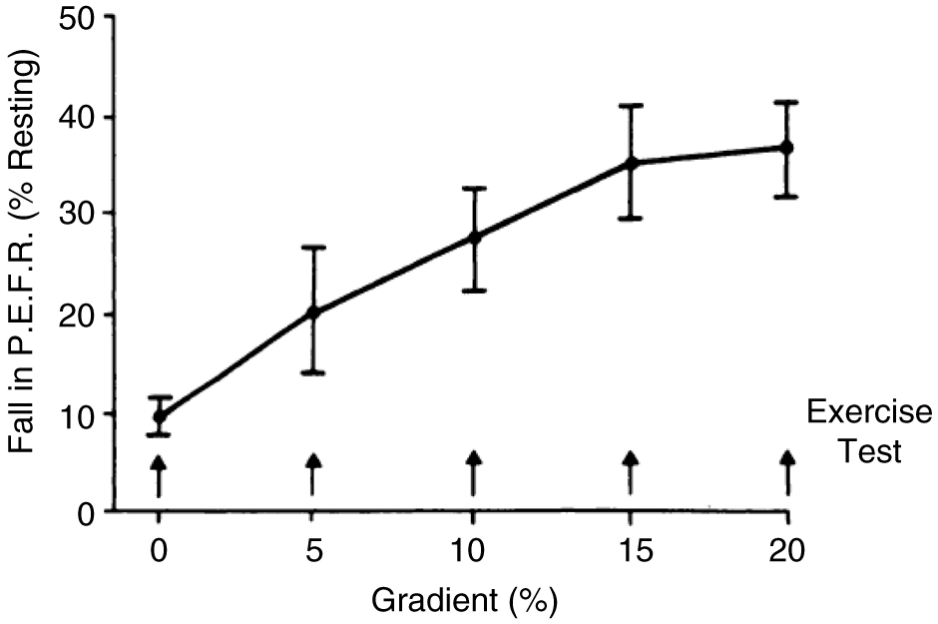
Effect of gradient (work load) on asthma, induced by treadmill running, at a constant speed for 6 min. Each point represents the mean of tests in nine subjects who performed each gradient on a separate day. The bars indicate±SEM. Reproduced with permission from (14).

**Fig. 3:**
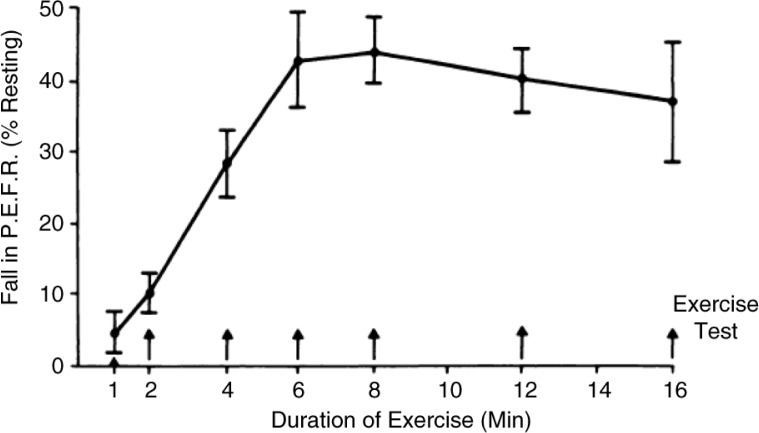
Effect of duration of exercise on asthma, induced by treadmill running, at constant speed and slope. Each point represents the mean of tests in 10 subjects who performed each duration on a separate day. The response plateaued at 6–8 min. The bars indicate±SEM. Reproduced with permission from (14).

**Fig. 4:**
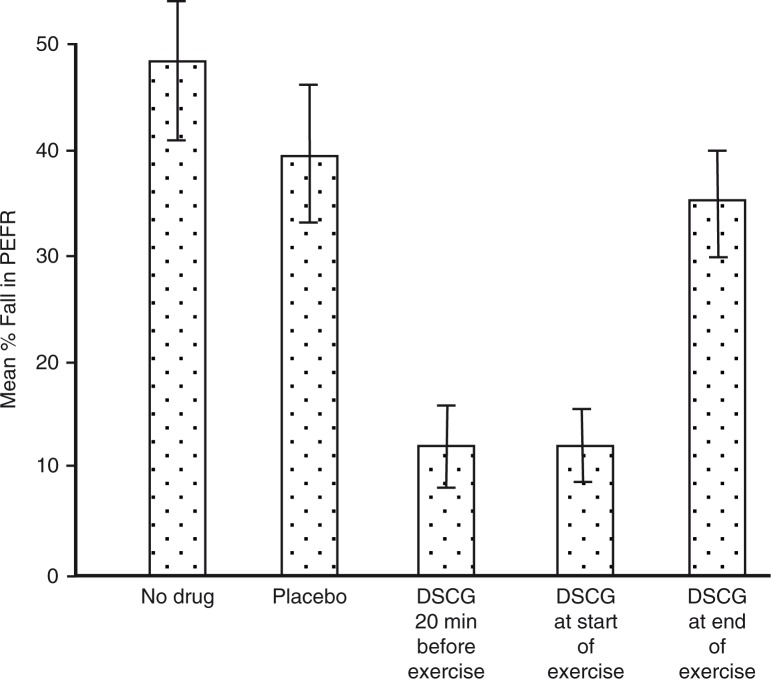
Disodium cromoglycate, taken 20 min, and at the start of running exercise and at the end of exercise compared with response after placebo and on a control day. The bars indicate±SEM. Adapted from (22).

In addition to type of exercise (Fig. 1), the intensity (Fig. 2), and duration (Fig. 3) of the running exercise were also found to be important, as was the interval between repeated tests (14, 21). By 1973, Godfrey et al. (14) in the UK concluded that ‘the greatest amount of exercise-induced asthma is found after 6–8 min of steady-state running at a work rate equivalent to two thirds of work capacity. Exercise may be repeated every 2 h throughout the day without any diminution of its causing post-exercise bronchoconstriction.’ This ‘standardized’ test provided a model to test the efficacy and duration of action for DSCG in children (14). Importantly, the drug was shown to be effective when given at the start of exercise (Fig. 4) and to inhibit EIA even in those who were non-atopic (22, 23).

A summary of research in EIA was published in 1974 following a symposium in Seattle, WA, USA. The contributors reported that EIA was most efficiently identified by measuring a fall in PEF or FEV_1_ of either 10% or 15% of the pre-exercise value (16, 24–26). The cycling was confirmed as poorly sensitive to identify EIA although it had some technical advantages (27). The use of a motorised treadmill was confirmed, and 6 min of running up a gradient of 10% at 5 km/h was recommended with the best reproducibility being obtained by repeating the test within 1 week (26). Free range running was also recognised as a useful test with the majority of those responding doing so within 5 min of ceasing exercise (28). In 1975, a 15% fall in FEV_1_ was identified as abnormal (24) though later studies identified a 10% cut off to define EIA (29).

While standardized protocols for exercise tests in children were published in the UK in the mid-1970s (14, 21, 30), the first clinical guidelines in the USA for testing both in adults and children were published in 1979 (31). These guidelines for adults proposed that the minimum equipment should provide for a continuous strip chart record of an ‘electrocardiogram’ and a stepped (progressive) protocol be used for the initial testing. Suggestions were also made for the steady-state exercise test of 5–8 min to be preceded by 4 min of increasing the speed and incline of the treadmill to reach the required heart rate (90% predicted maximum) and oxygen consumption (30–40 ml/min/kg) (32).

### Influence of humidity

Over the years, some investigators had noted that there was a seasonal variation in severity in some subjects and it was suggested that ‘changing patterns of humidity, temperature and wind velocity’ maybe responsible for increased susceptibility to EIA (28, 33).

In 1976, Weinstein et al. (34) at an Academy of Allergy meeting in Puerto Rico reported inhibition of EIA in 10 of 13 subjects after inhaling an aerosol of ultrasonically nebulized normal saline via a mask during running exercise. This observation was quickly followed up by many groups wanting to confirm this finding using water, inspired as a gas, rather than normal saline as an aerosol.

**Fig. 5:**
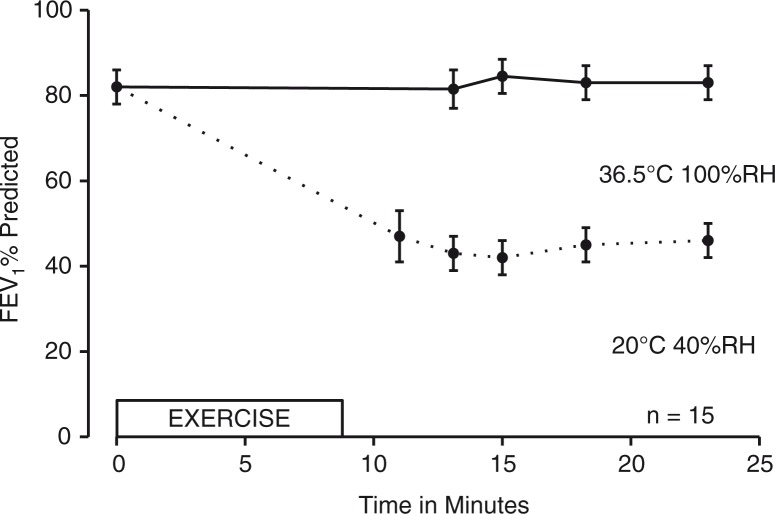
Mean±SEM values for FEV_1_ expressed as percentage of predicted before and after 5.5–8 min of treadmill running in 15 patients with severe EIA. Exercise was performed on two occasions 20 min apart with warm humid air as the first challenge (% fall 6.5±5.2 SD) and room air as the second (% fall 53.9±11.5 SD) (38). Figure reproduced with permission from (49).

In 1977, Bar-Or et al. (35) had children run in a climate chamber at 25–26°C with high (90%) or low relative humidity (25%). The mean fall in FEV_1_ was 36.8% in the dry air and this was reduced to <10% in the humid condition. They were the first to highlight ‘the importance of monitoring and standardizing the climatic conditions in the laboratory’ (35). Later in 1977, Chen and Horton (36) used inspired air at body conditions (37°C and 100% RH) to inhibit EIA. They found full protection from EIA in four asthmatic subjects who had a >20% fall in FEV_1_ after walking exercise inhaling dry air 23°C 15% RH. They concluded that EIA ‘must relate to the loss of heat/or water from the respiratory tract during exercise’ (36). The inhibitory effect of inspiring air at body conditions was confirmed by others studying cycling (37) and running exercise and including subjects with severe EIA (Fig. 5) (38).

### Heat loss and water loss: thermal load or osmotic load

Attention then turned to the effects of inspired temperature as a determinant of the response to exercise. In 1977, Strauss et al. (39) reported enhancement of EIA when cold air at −8 to −15°C was inspired during 3–4 min of ‘exhausting leg work on a cycle ergometer’. In 1978, using the same exercise protocol, they reported the effects of inspiring air at ambient and body temperature when fully saturated and when relatively dry. They showed that water content of the inspired air was the major determinant of severity of EIA (37). Including the data from these two studies, the same group proposed that the ‘magnitude of EIA is directly proportional to the thermal load’ and can be measured in terms of respiratory heat exchange (40). Furthermore, they suggested that ‘the major stimulus for EIA is heat loss with subsequent airway cooling’ (41).

By 1982, Anderson et al. had also studied the effect of varying heat and water content of the inspired air (42). In contrast to Deal et al. (40), they found that asthmatics varied by up to a factor 3 in their sensitivity to the thermal load even when correcting for lung size. They confirmed the greatest severity of EIA when subjects inspired dry air and the least with humid air. An unexpected finding was that 12 subjects still had EIA after inhaling air of body conditions (37°C 44 mg H_2_O/L) during and after exercise. Knowing that excess water could provoke bronchoconstriction (43), they concluded that ‘the bronchoconstricting effect from water gain and water loss from the airways may be a change in tonicity of the fluid lining the respiratory tract’ (42). Similar findings were reported in 1985, from a study using voluntary hyperpnoea (44).

**Fig. 6:**
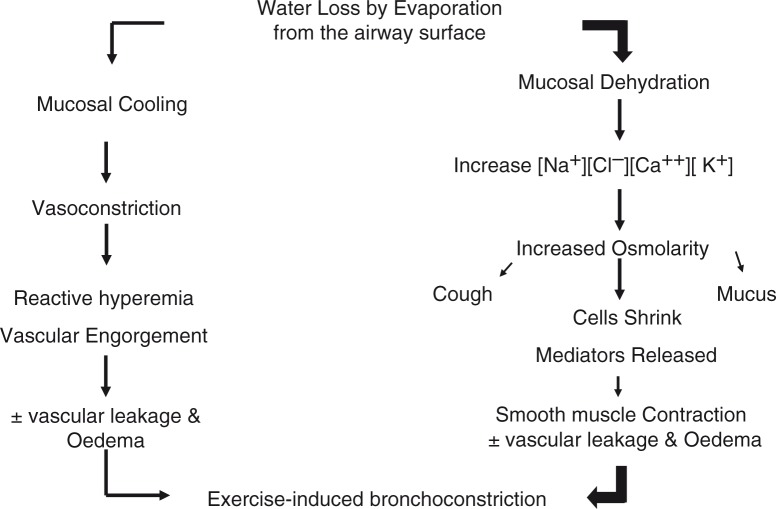
Evaporative water loss from the airways can lead to bronchoconstriction via airway cooling and rewarming and/or by airway dehydration and an increase in osmolarity of the airways. Dehydration is important for all temperatures of dry air, whereas cooling and rewarming will be additionally important when exercise is performed in subfreezing air conditions but become less so as warmer air is inspired. Reproduced with permission from (66).

The 1982 study of Anderson et al. (42) had not included an exercise test with a low inspired air temperature because no enhancement of EIA had been found when cool air was inspired in a pilot study. A casual comment to a colleague, about this unexpected finding, led to the publication of data from a climate chamber study using air of 9°C and 36°C but the same inspired water content (9–10 mg H_2_O/L) (45). The severity of EIA was the same under both conditions where water loss, but not heat loss, was the same. It was argued that ‘the osmotic and not the cooling effects induced by vaporization of water was the important factor determining EIA’ (45). This proposal was presented in detail as a unifying hypothesis for EIA (46). The conclusion was that ‘there may be an important association between osmotic changes in the epithelium and the release of mediators from bronchial mucosal mast cells’ (Fig. 6) (46).

To investigate further the airway cooling hypothesis, a study of the effects of hot dry air (32–40°C) was performed in children cycling for 8 min (47). Moderate-to-severe EIA (mean% fall FEV_1_ 39.8%±22.3) occurred in 20 of the 22 subjects and the expired air temperature during exercise was higher than at rest (35.3°C versus 33.2°C) (47). This finding demonstrated that abnormal airway cooling was not occurring and thus not essential for EIA to occur. They proposed ‘water loss and not heat loss was the stimulus to EIA under these inspired air conditions’ (47). A similar conclusion in respect to respiratory heat loss was made by others using voluntary hyperpnoea (48).

The water per litre of expired air was found to be relatively constant between 31 and 33 mg/L when air over a wide range of temperature was inspired at 22–40°C (49). Providing the ventilation was high enough for long enough, then the rate and amount of water loss would be sufficient to provide an osmotic load. It was not feasible, however, to obtain a representative sample of surface liquid from the lower airways during exercise. That evaporative water loss could increase airway osmolarity was confirmed in the human nose (50). Mathematical models estimated that exercising under temperate conditions, 40% of the water lost would come from the lower airways (51).

### Air condition during recovery and thermal gradients

In 1986, McFadden et al. (52) reported that the condition of the air inspired during recovery from exercise determined the magnitude of airway response in adults. They found that exercising for 4 min breathing cold air followed by 5 min breathing air of body conditions during recovery resulted in a greater fall in FEV_1_ in both asthmatic and normal subjects (52). They concluded that ‘in order to induce obstruction a thermal gradient seems to be necessary at the end of challenge so that the cooling brought about by hyperpnea is followed by rewarming when hyperpnea ceases’ (52). While airway cooling followed by rapid rewarming could amplify the airway response to water loss particularly in cold weather athletes (53), it could occur independently of the osmotic effects (Fig. 6) (54). A thermal gradient did not appear essential (55) because EIA occurred when breathing hot (≥36°C) dry air both during and after exercise when a thermal gradient would be minimal if not absent (37, 40, 45, 47, 56). Further flow rates often fall during exercise of 6–8 min duration and before any rewarming could occur (56). Finally, the observations (52) were not reproduced in children who exercised for 5.5–10 min (57). The laboratory protocols did not include air conditions during recovery from exercise, but they did recommend 6–8 min of vigorous exercise, rather than 4 min. The longer duration was known to enhance the magnitude of the airway response under both temperate and hot air dry air conditions (21, 56).

### Defining the protocol for clinical practice

Once all the important factors determining the airway response to exercise were identified from research studies, protocols were established for use in clinical practice (58–60). In essence, these stated that to identify EIA it required the duration of exercise be 6–8 min and the exercise load, either cycling or running, be of sufficient intensity to raise ventilation to 40–60% of maximum voluntary ventilation (MVV), where MVV equals 35 times FEV_1_. This intensity was to be maintained for 4 min and the inspired air needed to contain <10 mg H_2_O/L. The nose was to be clipped and a suitable time period to have elapsed since last medication or vigorous exercise. The heart rate was required to be measured continuously and, in those over 40 years, an electrocardiogram was taken throughout exercise and for 5 min after its completion.

Over the years, heart rate became a surrogate for ventilation, as a measure of intensity of exercise. This outcome was unfortunate in that heart rate during exercise does not reflect ventilation or rate of respiratory water loss (61). The major determinants of EIA remain the level of ventilation reached and sustained during exercise and the water content of the inspired air.

**Fig. 7:**
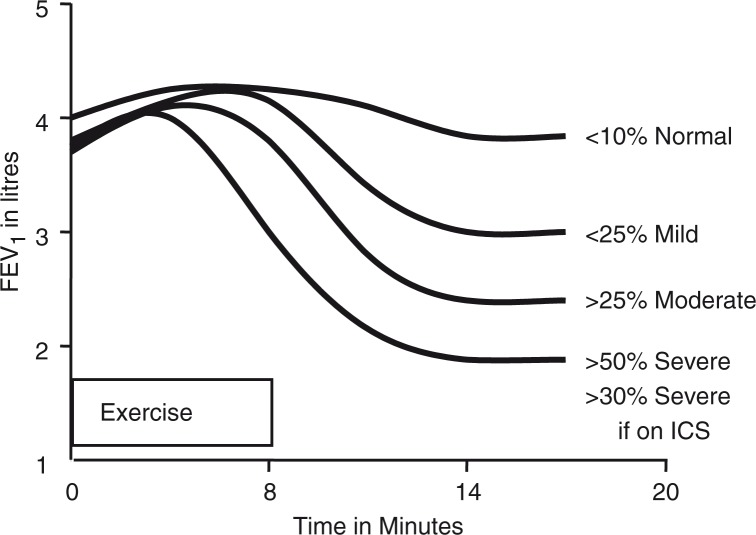
Pattern of change in FEV_1_ after 8 min of vigorous exercise inspiring dry air at a ventilation exceeding 50% of maximum, in a normal healthy subject without exercise-induced bronchoconstriction (EIB) and in subjects with mild, moderate, or severe EIB. The severity of the response is based on the maximum fall in FEV_1_ in the 20 min after exercise expressed as a percentage of the baseline value. If a subject is taking inhaled corticosteroids on a daily basis, a post exercise fall in FEV_1_ of 30% or more would be considered severe. Reproduced with permission from (66).

There has been some ‘fine tuning’ from subsequent studies. For example, a protocol for exercise in the field taking climatic conditions into account suggested a 15% fall in FEV_1_ to identify EIA in children (62, 63). A cut-off of a 13% fall in FEV_1_ was suggested by Godfrey after analysing published data in a ~1,000 normal children (64). Combining two indices of spirometry improved sensitivity to identify EIA (65). Levels of severity for EIA were suggested (66) (Fig. 7) and later adopted (67). The greater sensitivity of exercising, in the field, rather than in the laboratory was emphasised (68). The importance of ensuring that exercise load is appropriate in children and heart rate is preferably 95% during the last 4 min of exercise has been highlighted (69). The FEV_0.5_ was introduced to assess EIA in young children (70). The reproducibility of the response to a running exercise protocol demonstrated the need for two negative tests before EIA could be confidently excluded (71). A novel test to identify EIA in very young children was introduced involving exercising on a jumping castle in a cool environment (72).

Recommendations and protocols to identify EIA in clinical practice have been published by both the European (73) and American Academies of Allergy and Clinical Immunology (74) and the American Thoracic Society (67).

### Eucapnic voluntary hyperpnoea of dry air

In the clinical laboratory, it became difficult to ensure that all subjects, under investigation for EIA, could actually exercise on a bicycle or a treadmill to a sufficient intensity and sustain a level of ventilation for long enough to provoke EIA and there was a high frequency of negative tests. These problems plus the safety measures required by guidelines to study adult subjects likely contributed to the disenchantment with exercise testing and enhanced the development of a surrogate test for identifying EIA.

**Fig. 8:**
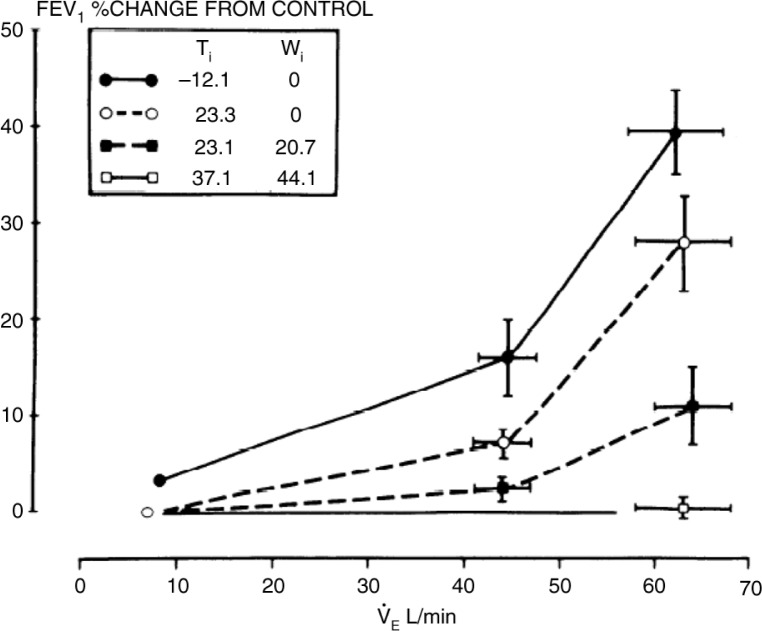
Changes that developed in L/sec in FEV_1_ at various levels of ventilation (V_E_) and various inspired air conditions for temperature and water content (see inset). Values at extreme left of the graph represent effects of inhaling each gas mixture on lung function at rest as determined from previous studies in the same subjects. Reproduced with permission from (41).

Studies in both children and adults (75–77) indicated that a major determinant of the severity of EIA was not the exercise itself but the rate of ventilation achieved and sustained during exercise. In 1979, Deal et al. (41) had reported equivalent bronchoconstriction with both exercise and hyperpnoea of dry air over a wide range of air conditions. These findings clearly demonstrated that humoral substances released during exercise were not relevant, and that ventilation and inspired air condition (Fig. 8) could explain much of the difference in severity of EIA between different forms of exercise (41). Thereafter, hyperpnoea with dry air presented an attractive option to identify EIA in that it reduced the cost of equipment substantially and obviated the need for trained personnel to supervise an exercise test.

In the early 1980s, a number of protocols were developed using voluntary hyperpnoea of dry air with added carbon dioxide (CO_2_) as a surrogate for exercise-induced hyperpnoea (78, 79). The CO_2_ was introduced in sufficient quantities to keep end-tidal CO_2_ (P_ET_CO_2_) levels within the normal range. There were two major differences between the types of protocol developed. One difference was the temperature of the inspired air, either air at room temperature or air conditioned to subfreezing temperatures. The other difference was choice of either a single level of ventilation or multiple levels that progressively increased.

A cold air (−15°C) challenge with ventilation (V_E_) increasing every 3 min (7.5, 15, 30, and 60 L/min) was developed by O'Byrne et al. (80). They reported the value for respiratory heat exchange (RHE) that provoked a 20% fall in FEV_1_. This protocol was also used to study the inhibitory effects of DSCG (81). In contrast to O'Byrne et al., Aquilina (82) chose a single level of V_E_ of 24×FEV_1_ L for 3 min and used both room temperature air and air at −10°C with the cold air causing a fall in FEV_1_ in all subjects that was >9% of the resting value.

The RHE index was impractical and hard to interpret for most investigators and after using the same protocol, the more simple index of ventilation to provoke a 20% fall in FEV_1_ was suggested by Weiss et al. (83). Others found that repeatability of the cold air test, as with exercise, was better between days rather than within a day (84). The protocol with progressive increases in ventilation was included in the 1993 document on standardized lung function testing (58).

Another protocol included in the same document (58) was a voluntary hyperpnoea test for children standardized for different ages and sizes (85). Zach et al. used a single level of ventilation equivalent to 75% MVV for 4 min at −17°C and reported changes in FEV_1_ as the being the most reproducible measurement (85). This protocol was used for many years (86) including in a survey of 5,697 children in Germany (87). It was later modified for use in very young children (2–5 years) with specific airway resistance being measured by whole body plethysmography, and the test was recommended to identify asthma (88).

All the protocols necessitated breath-by-breath monitoring of the P_ET_CO_2_ with a rapid gas analyser and for many the conditioning of inspired air to subfreezing temperatures. Although several commercial devices became available to generate cold air at −10°C, they required excessively high volumes of compressed air adding to the cost of the test. These technical requirements made these protocols for voluntary hyperpnoea unattractive for use in clinical laboratories and a more simplified system was needed.

The simplified system was provided by using a constant level of CO_2_ in a gas mixture delivered at room temperature (89, 90). When the inspired air contained 4.89% CO_2_ and the FEV_1_ was >1.5 L, the P_ET_CO_2_ remained in the eucapnic range of ~38–42 mmHg at ventilations between 40 and 105 L/min. Using this method, the changes in FEV_1_ following challenge were shown to be similar to that provoked, in the same subjects, by exercise at the same ventilation. It was called eucapnic voluntary hyperventilation and was really developed as a bronchial provocation test to identify EIA in potential recruits to the defence force. Over a series of studies, it was reported that 1) a 10% fall in FEV_1_ 5–10 min after eucapnic voluntary hyperpnoea (EVH) was an asthmatic response (91). 2) The dosing schedule should be standardized at a single level of uninterrupted ventilation (92, 93). 3) Dry air was more sensitive to identify AHR than cold air and much more sensitive than cycling exercise, and 4) a fall in FEV_1_ >10% of the resting value after 5–6 min of hyperpnoea at 60–80% of MVV was diagnostic of AHR (94).

A higher level of ventilation (30 times FEV_1_) equivalent to 85% MVV was also suggested with the defence force recruits in mind. This ventilation is higher than the 50–65% MVV achieved during exercise by non-trained subjects and much closer to that achieved by elite athletes. For this reason, an update of the original protocol described by Argyros (92) was published (95) and recommended for identifying EIA in elite athletes for the Winter Games in Salt Lake City (2, 96, 97).

**Fig. 9:**
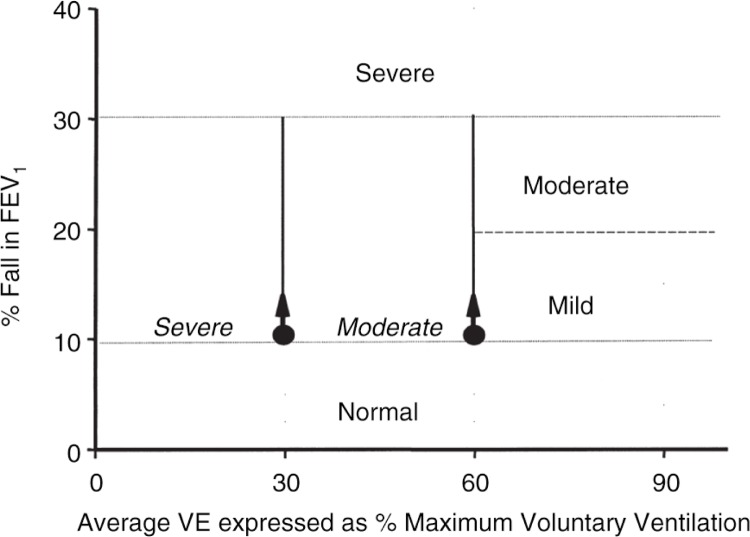
The classification of severe, moderate, and mild is made on the level of ventilation required to induce a positive response. For example, ≥10% fall in FEV_1_. The response is severe if a positive response is obtained at 30% maximum voluntary ventilation (MVV), moderate if it is positive at 60% MVV, and mild if it is positive at 90% MVV. A fall in FEV_1_ greater than 30% whatever the ventilation would be regarded as severe. This plot can be used for a multistage or single-stage test. Maximum voluntary ventilation can be calculated on the predicted or actual FEV_1_ with MVV=35×FEV_1_. By relating the ventilation to a common predicted value, values between subjects can be compared. This also allows the intensity of exercise inducing the response to be estimated. Reproduced with permission from (2).

This test became known as the EVH test. It required a source of compressed gas at room temperature containing 21% O_2_, 4.9–5% CO_2_, and the remaining N_2_, and the subject was required to breathe this for 6 min at a single level of ventilation equivalent to 30 times the FEV_1_ (2, 92, 95).The severity of the response is described in Fig. 9, (2). A population of elite athletes was tested and the EVH found to be the best to identify potential for EIA (98, 99) and the test continued to be recommended for assessment of elite athletes (100).

The required duration of EVH is shorter than exercise because the target ventilation is reached in seconds rather than minutes as occurs with exercise (61). The rate of water loss at target ventilation needs to exceed water replacement so that, over time, more airways are recruited into the conditioning process. A big advantage of EVH is that the target ventilation exceeds the maximum ventilation achievable during exercise so that false-negative tests are unlikely. In research studies of known asthmatics, however, it was recognised that the high level of ventilation and the duration of 6 min had the potential to cause large unwanted falls in FEV_1_. For this reason, the dosing protocol was modified to evaluate the effects of drugs (101, 102) to compare with other stimuli (103) and to assess subjects with symptoms of asthma in a clinic (104, 105). A valid clinical test for EIA is considered to be one where the ventilation reached and sustained is 60% of MVV or 21 times FEV_1_ (104) and is the one recommended for challenging known asthmatics (95).

In the last 10 years, a delivery system capable of monitoring and adjusting CO_2_ to maintain eucapnia during hyperpnoea up to 220 L/min, with a display screen, became commercially available (Eucapsys, SMTEC, Switzerland). This facilitated identification of EIA in athletes in the clinical laboratory because the ventilation was not limited to 105 L/min as was necessary when a constant level of 4.9–5% CO_2_ concentration was inspired. Other devices have also been used (Ailos Medicinsk Teknik, Karlstad, Sweden) and another recently developed in the USA (Rosenthal Hyperventilometer).

### Aerosols of hypertonic saline

The idea to perform a challenge with an aerosol of hypertonic saline arose as a result of the report of the bronchoconstricting effects of inhaling an aerosol of water by Allegra and Bianco (106). They had used an ‘ultrasonic mist of distilled water’ delivering 2 ml/min via a face mask and reported a significant increase in specific airways resistance in asthmatics, but not normal subjects. No response occurred following the inhalation of normal saline. They reported the inhibitory effect of DSCG and proposed that mast cell release of histamine was consistent with the airway response (107–109).

Using the same nebulizer (MistO_2_gen EN 143 Timeter PA), Schoeffel et al. (43) administered increasing doses of both hypotonic and hypertonic saline aerosols and measured the airway response using FEV_1_ rather than airways resistance. The aerosol was inhaled via a Hans Rudolph 2700 valve, and the expired ventilation was measured using a Drager volume meter. Ten asthmatic subjects with EIA were studied. Initially, 5 or 10 L of the aerosol was inspired through the nebulizer and the FEV_1_ measured 30 sec later. When the fall in FEV_1_ was <10% of baseline, the volumes used in subsequent exposure were 20, 40, 80, 80, and 80 L until a 20% fall in FEV_1_ had occurred or 310 L had been inhaled. The airway response was also expressed as the volume to provoke a 20% fall in FEV_1_ from baseline (PV_20_). Schoeffel et al. (43) confirmed the earlier findings with distilled water and isotonic saline and were the first to report the bronchoconstricting effects of hypertonic aerosols of saline (2.7% and 3.6%) in subjects with asthma. They stated that the effect was likely due to osmolarity as inhaling an aerosol of 20% dextrose provoked similar changes in FEV_1_ (43). Citing that both hypo and hypertonicity resulted in release of histamine from mast cells (109, 110), they proposed that water movement in and out of the mast cell was the stimulus for mediator release. They concluded that ‘the method used for the challenge was rapid, simple and inexpensive and provides a new technique for the diagnosis of non-immunologically mediated bronchial hyperreactivity’ (43).

In the early studies, the challenge with hypertonic saline started with a 60-sec exposure and the test continued until a 20% fall in FEV_1_ or 30 ml had been delivered. Many asthmatic subjects were very sensitive to these aerosols and the initial exposure time was reduced to 30 sec and the maximum dose to 15.5 ml. The dose of aerosol delivered by the ultrasonic nebulizer was found to be constant, independent of air flow and directly related to expired volume so that time could also be used for a dosing schedule. Exposure times were 30 sec 1, 2, 4, and 8 min with FEV_1_ being measured in duplicate 60 sec after each exposure. The use of time made the method practical for use in clinical practice. The nebulizer unit with accompanying tubing, but not the valve, was weighed before and after challenge to calculate the total dose of aerosol delivered, and a dose–response curve was constructed. In 1983, the provoking dose of water or hypertonic saline to induce a 20% fall in FEV_1_ (PD_20_) replaced the (PV_20_). As the majority (80%) of asthmatic subjects responded in <9 min, this made the protocol feasible as a routine provocation test even though a minority of subjects required 20–25 min to respond. Consistent with exercise and hyperpnoea with cold air, the responses to both water and 3.6% saline responses were inhibited by DSCG (111). The method was published in detail in 1984 and 1985 (112–114).

Both hypo and hypertonic aerosol challenge tests were included in the Sterk document in 1993 (58). The distilled water test was used extensively for research, particularly for assessment of drugs (115–117). There were a number of findings however that probably contributed to it never becoming established in clinical practice. These included the finding it caused excessive cough and increased non-specific AHR (118–120). Further, it was found that the presence of permeant anions reduced cough making other aerosols more attractive (121).

**Fig. 10:**
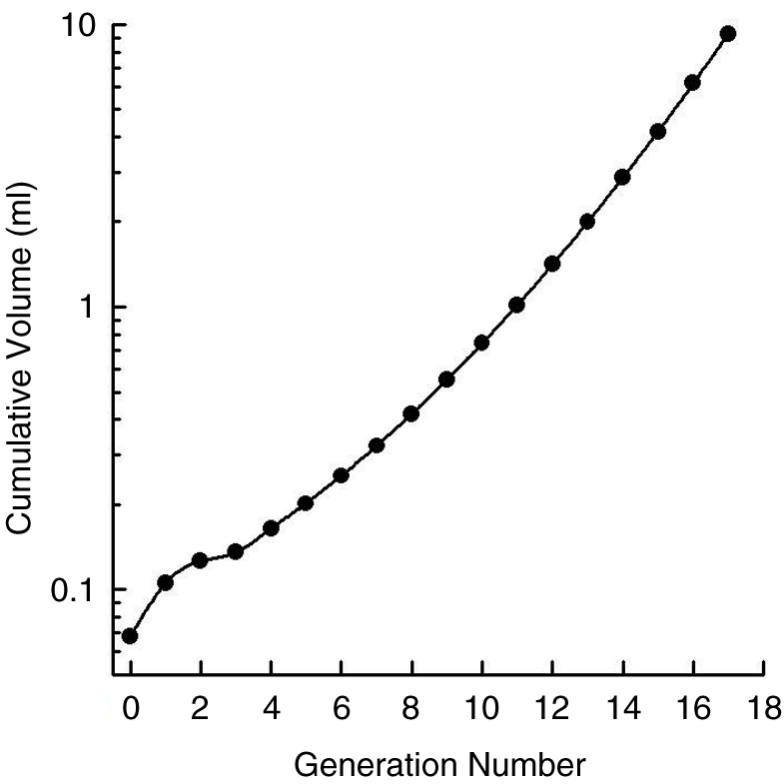
The cumulative volume of airway surface liquid, in relation to the number of generations of airways assuming 10 µm for the periciliary fluid depth. Reproduced with permission from (123).

The development of the challenge with hypertonic saline aerosol continued because of its potential to mimic the osmotic effects of evaporative loss of water from the airway surface, a stimulus proposed to account for EIA (Fig. 6) (45, 46). This potential was shown to be realistic in 1983 when computer-generated calculations based on the airway dimensions of Weibel (122) revealed that the cumulative surface area of the first seven generations of airways was <400 cm^2^ (56) and the cumulative volume correspondingly small (Fig. 10) (123). For example, the cumulative volume of airway surface liquid for seven generations was estimated at 0.39 ml (46, 56, 124). From that calculation, it was immediately obvious that only a very small volume of water needed to be lost by evaporation during exercise, or a very small volume of hypertonic saline needed to be deposited on the surface of these airways to cause a marked increase in osmolarity of the airway surface.

**Fig. 11:**
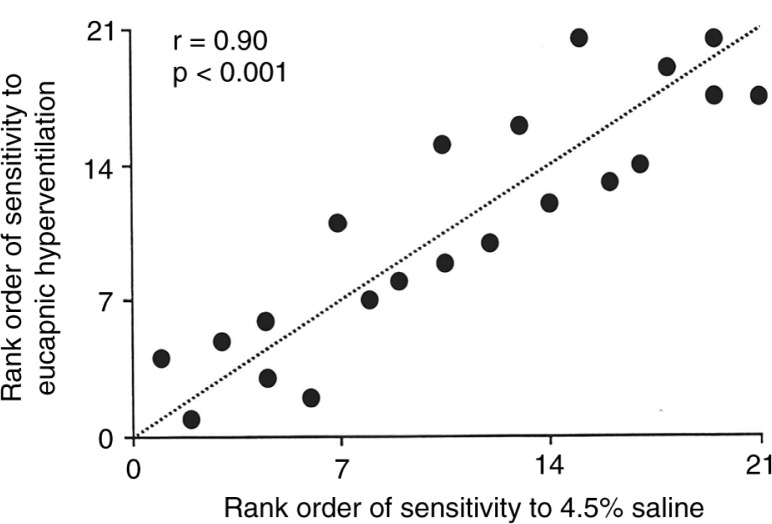
The Spearman's rank correlation illustrates the relationship between the sensitivity to eucapnic hyperpnoea with dry air and sensitivity to 4.5% saline in 21 subjects. Reproduced with permission from (124).

**Fig. 12:**
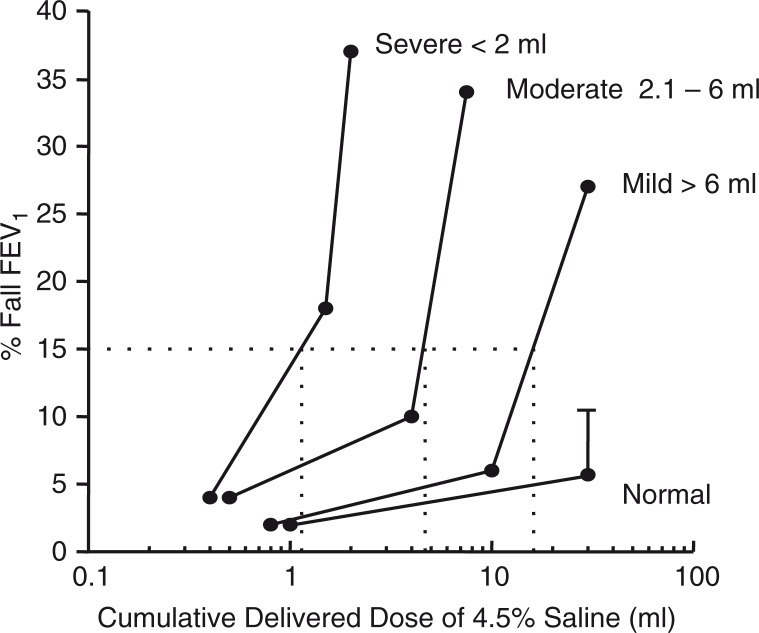
Classification of the response to hyperosmolar to 4.5% saline in terms of the provoking dose of aerosol required to induce a 15% fall in FEV_1_. The delivered dose is cumulative and is calculated by dividing the total dose delivered over the time of the challenge. For normal subjects, the mean plus 1 SD is shown. Reproduced with permission from (2) with data for normal subjects from (156).

Continuing investigations, with different concentrations of saline, demonstrated it was rate of change of osmolarity that was important so concentration was increased from 3.6% (concentration of sea water) to 4.5% (125). The increase to 4.5% saline reduced the exposure time and the chance of false-negative tests and made the test practical for clinical use (126). Comparisons were made with exercise and EVH, and the sensitivity of subjects to the tests found to be concordant in most cases (Fig. 11) (124, 127–129). As inhaled corticosteroids were becoming more commonly prescribed at the time, there was interest in using a challenge test that had a high specificity to identify currently active asthma. Subsequently, the finding that sensitivity to 4.5% saline was linearly related to the % of mast cells obtained from brush biopsy of the airways (130) and it was reduced by treatment with ICS (131, 132) contributed to its adoption for clinical use (124, 133). The availability of data in healthy subjects resulted in a positive response to 4.5% saline test being reduced from 20% to 15% (Fig. 12) (2, 124). It was also an advantage that sputum could be harvested to assess cellular count at the same time (134, 135).

Challenges with hypertonic saline aroused interest, not only from hospital-based clinicians but also from the defence forces, the underwater diving fraternity, occupational physicians, paediatricians, and epidemiologists. Suitability to dive was assessed in those with a past history of asthma (136). The hypertonic saline was used for assessing suitability for mild asthmatics to join the defence forces (137). The same protocol was used in a field survey in an occupational setting (138). In a study in children those who were positive to hypertonic saline were five times more likely to have EIA (139). The hypertonic saline became well characterised for use in children (140–142) and used along with inflammatory markers to identify asthma in children (143).

### Dry powder aerosol of mannitol

By the mid-1990s, it was obvious that there were technical and hygienic limitations in generating dense aerosols from ultrasonic nebulizers in the laboratory environment. The test required filters and scales for weighing, and cleaning procedures took time and were cumbersome. Further, the particle size of the aerosol could change over the life of the piezoelectric crystal of the nebulizer. To simplify the use of hypertonic saline for identifying AHR, a dry powder was developed. Both sodium chloride (144) and mannitol were trialled (145). Mannitol was selected because it was a naturally occurring substance generally regarded as safe, commonly used as an excipient, stable at high levels of humidity and not absorbed to any significant extent by the gastrointestinal tract. Mannitol was known to stimulate the release of histamine from human lung mast cells *in vitro* (146). Importantly, the release of histamine was enhanced in the presence of anti-IgE and optimal at 32°C. The release of histamine occurred after an exposure to the hyperosmolar solution of only 60 sec (146, 147). Further, this release could be blocked by DSCG (148).

**Fig. 13:**
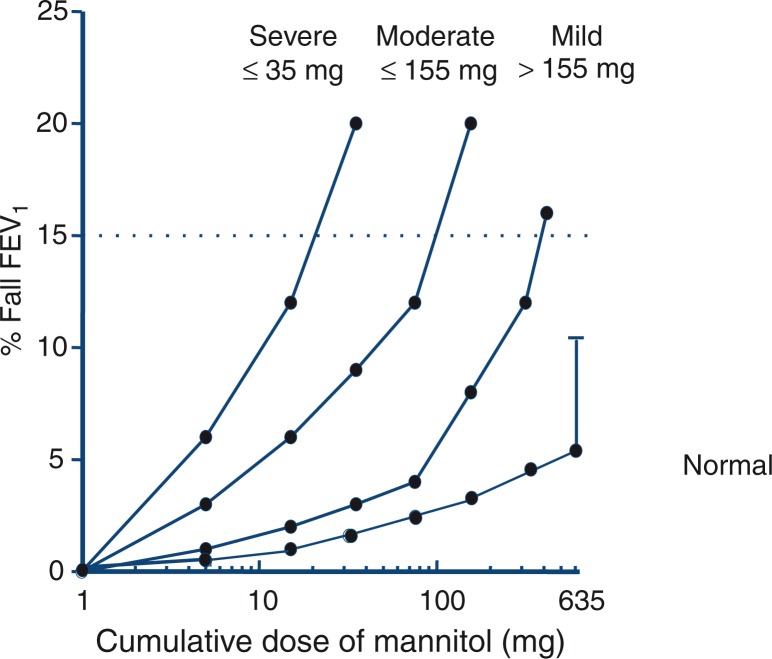
Classification of the response to mannitol in terms of the provoking dose of powder delivered from the capsules required to induce a 15% fall in FEV_1_ (PD_15_). For normal subjects, the mean plus 1 SD is shown. Reproduced with permission from (2), with data for normal subjects from (156).

The mannitol powder was prepared by spray drying and was encapsulated and delivered in doses (5, 10, 20, 40, 80, 160, 160, and 160 mg) from a disposable dry powder inhaler. FEV_1_ was measured 60 sec after each dose and a 15% fall in FEV_1_ after inhaling 635 mg or less was taken as indicative of a positive response (Fig. 13) (2, 145). The sensitivity to mannitol was expressed as a PD_15_ and reactivity as the response–dose ratio (RDR), that is,% fall in FEV_1_ at the end of challenge divided by the cumulative dose of mannitol that achieved the fall. This index permitted all tests to be analysed, whether a PD_15_ was obtained or not, so it was useful to assess the beneficial response to inhaled corticosteroids (149).

A series of clinical studies in Australia, Canada, Finland, and Switzerland established the potential for mannitol to become a simple point of care test to identify AHR consistent with currently active asthma and airway inflammation responsive to inhaled corticosteroids (145, 149–155). The intellectual property, owned by the public health service, was licensed to a pharmaceutical company. Two Phase 3 trials (156, 157) were carried out in two populations, one with an established diagnosis of asthma and the other in subjects with symptoms of asthma but no definite diagnosis. The Phase 3 trials for registration of mannitol required comparison with other tests, one being with the wet aerosol of 4.5% saline, (156) another running exercise and another methacholine chloride (157). The time to document a positive response to mannitol was 17.3 min and a negative response 26 min (156). The time for challenge and the high specificity (95%) to identify asthma made the mannitol test attractive to clinicians particularly in relation to decision to treat with ICS (156, 157). The false-negative tests contributing to a sensitivity of 60% occurred in those currently being treated with ICS or in subjects with mild asthma not requiring ICS (156, 158). When the use of steroids was taken into account, the sensitivity to identify asthma using the mannitol test rose to 89% (156).

The mannitol test received regulatory approval in Australia in 2006, the European countries between 2006 and 2008, and Korea and USA in 2010. The mannitol test kit of capsules and inhaler (known as Aridol™ or Osmohale™ Pharmaxis Ltd, Frenchs Forest, NSW, Australia) provides a common operating procedure for bronchial provocation across the globe and requires only the minimum of equipment including a spirometer and clock. The test was taken up into clinical practice and included in guidelines (67, 74, 159).

Since registration there have been many studies assessing mannitol responsiveness as both a diagnostic test to identify EIA (160–162) and a clinical diagnosis of asthma (163, 164). It has been compared with inflammatory biomarkers of asthma (165, 166). Several large studies using inhaled steroids have demonstrated usefulness for both down titrating (167) and up titrating dose based on AHR to mannitol (168).

### Characteristics of all indirect challenges

The characteristics that make challenges with stimuli, that act indirectly, different, and potentially more useful in clinical practice than stimuli that act directly (e.g. methacholine) are summarised as follows. All the responses to indirect challenge tests 1) have a high specificity to identify people with current asthma (156, 157, 163, 164), 2) are inhibited by chronic treatment with inhaled corticosteroids (132, 149, 155, 169–177), 3) are inhibited by acute treatment with a leukotriene receptor antagonist or a 5-lipoxygenase inhibitor (154, 178–183), 4) are associated with the release of the specific mast cell mediator PGD_2_ and release of leukotriene E_4_ (184–191), 5) are inhibited by acute treatment with DSCG (81, 132, 188, 190, 192–195), and 6) are subject to a refractory period that is usually <3h (93, 196–201).

## Conclusion

The scientific research that identified the stimulus to EIA, as evaporative loss of water from the airway surface, was fundamental to the development and standardization of these indirect challenge tests. The safety of these tests was established by their successful use in large numbers of subjects in Phase 3 trials, clinical trials, and in field studies. All these challenge tests have a high specificity to identify currently active asthma. All can be used to identify the need for treatment and for the assessment of response to inhaled corticosteroids and other anti-inflammatory drugs. Over the 50 years, the methods for testing became less complex, requiring less expensive equipment, using an easy measure to express sensitivity and reactivity. Thus, they became practical to use routinely and were recommended in guidelines for use in clinical practice.
